# Cancer stem cell subpopulations in primary colon adenocarcinoma

**DOI:** 10.1371/journal.pone.0221963

**Published:** 2019-09-06

**Authors:** Matthew J. Munro, Susrutha K. Wickremesekera, Lifeng Peng, Reginald W. Marsh, Tinte Itinteang, Swee T. Tan

**Affiliations:** 1 Gillies McIndoe Research Institute, Newtown, Newtown, Wellington, New Zealand; 2 School of Biological Sciences and Centre for Biodiscovery, Victoria University of Wellington, Kelburn, Wellington, New Zealand; 3 Upper Gastrointestinal, Hepatobiliary & Pancreatic Section, Department of General Surgery, Wellington Regional Hospital, Private Bag 7902, Wellington, New Zealand; 4 University of Auckland, Grafton, Auckland, New Zealand; 5 Wellington Regional Plastic, Maxillofacial & Burns Unit, Hutt Hospital, Lower Hutt, New Zealand; National Institute of technology Rourkela, INDIA

## Abstract

**Aims:**

The cancer stem cell concept proposes that tumor growth and recurrence is driven by a small population of cancer stem cells (CSCs). In this study we investigated the expression of induced-pluripotent stem cell (iPSC) markers and their localization in primary low-grade adenocarcinoma (LGCA) and high-grade adenocarcinoma (HGCA) and their patient-matched normal colon samples.

**Materials and methods:**

Transcription and translation of iPSC markers OCT4, SOX2, NANOG, KLF4 and c-MYC were investigated using immunohistochemical (IHC) staining, RT-qPCR and *in-situ* hybridization (ISH).

**Results:**

All five iPSC markers were detected at the transcriptional and translational levels. Protein abundance was found to be correlated with tumor grade. Based on their protein expression within the tumors, two sub-populations of cells were identified: a NANOG^+^/OCT4^-^ epithelial subpopulation and an OCT4^+^/NANOG^-^ stromal subpopulation. All cases were accurately graded based on four pieces of iPSC marker-related data.

**Conclusions:**

This study suggests the presence of two putative sub-populations of CSCs: a NANOG^+^/OCT4^-^ epithelial subpopulation and an OCT4^+^/NANOG^-^ stromal subpopulation. Normal colon, LGCA and HGCA could be accurately distinguished from one another using iPSC marker expression. Once validated, novel combinations of iPSC markers may provide diagnostic and prognostic value to help guide patient management.

## Introduction

The cancer stem cell (CSC) concept hypothesizes that tumor growth is driven by CSCs, a small subpopulation of cancer cells with stem cell characteristics [1–4]. CSCs produce identical daughter pluripotent cells, as well as progenitor cells which are more committed and sit on a hierarchy between CSCs and terminally differentiated cancer cells [5–7]. Cells within this hierarchy can be identified by their expression of different combinations of markers [6, 8]. Tang [5] postulates that progenitor cells are responsible for uncontrolled growth.

Takahashi and Yamanaka [9] first used octamer-binding transcription factor 4 (*OCT4*, *POU5F1)*, sex-determining region Y-box 2 (*SOX2)*, Krüppel-like factor 4 (*KLF4)* and *c-MYC* to produce induced-pluripotent stem cells (iPSCs). The Thomson laboratory produced iPSCs from human fibroblasts using *OCT4*, *SOX2*, *NANOG* and *LIN28* [10].

OCT4 is a transcription factor involved in stem cell maintenance, and has been observed in normal colon stem cells [11]. Studies have shown OCT4 expression in colorectal cancer (CRC), often in the cytoplasm of epithelial cells [11–13]. The OCT4B isoform is unable to act as a transcription factor and is often localized to the cytoplasm, while the OCT4B1 spliced variant is over-expressed in high-grade CRC [14].

SOX2 maintains pluripotency of ESCs and neural progenitor cells and is critical for early embryogenesis [11, 15]. It is involved in regulating *OCT4* transcription by binding to its promoter region [16–18]. Studies have localized SOX2 to the cytoplasm and nuclei of both normal and cancerous crypt epithelial cells [19]. SOX2 expression is associated with lymph node infiltration and metastasis in CRC [20].

NANOG transcription is controlled by the OCT4/SOX2 transcription factor complex [16, 21]. NANOG has been detected in colon cancer and dysplastic polyps, often exhibiting strong nuclear staining in a subpopulation of epithelial cells within the crypts [11, 22].

*c-MYC* has been well-studied for its role as a proto-oncogene and is often over-expressed in cancer [9]. Duplication of the *c-MYC* gene is associated with a worse prognosis in CRC [23]. Therapy-naïve CRC cells with high c-MYC expression progress more quickly, and CRC metastases exhibit greater c-MYC expression than the primary tumor [24].

KLF4 is associated with colon sphere-forming cells and involvement in cell cycle, pluripotency and self-renewal [16, 25]. KLF4 is a marker of differentiation down the goblet cell epithelial lineage from intestinal stem cells [26]. In CRC, KLF4 levels decrease with increasing histological grade, with poorly-differentiated (high-grade) tumors expressing less KLF4 than well-differentiated (low-grade) tumors [27].

Primary colon adenocarcinoma (CA), the most common type of CRC, is categorized as low-grade (well- and moderately-differentiated tumors with greater than 50% crypt and gland composition) or high-grade (poorly-differentiated tumors with densely-packed tumor cells) [28]. Although CSCs have been previously studied in CRC, the putative subpopulations of CSCs are yet to be characterized. We hypothesized that CA contains subpopulations of CSCs which can be identified by their expression patterns of iPSC-related markers. In this study, we investigated the level of iPSC marker transcription and translation, and their distribution within the epithelium and stroma, to determine the difference between low-grade CA (LGCA) and high-grade CA (LGCA) and their patient-matched normal colon (NC), using 3,3-diaminobenzidine (DAB) and immunofluorescence (IF) immunohistochemical (IHC) staining, RT-qPCR, and *in-situ* hybridization (ISH).

## Materials and methods

### Tissue samples

Snap-frozen and formalin-fixed paraffin-embedded (FFPE) tissue samples of LGCA from ten patients and HGCA from eight patients, with patient-matched normal colon (NC) tissue samples from 17 of the 18 patients, were provided by the Gillies McIndoe Research Institute Tissue Bank for this study, which was approved by the Central Health and Disability Ethics Committee (Ref. 15/CEN/106).

### DAB IHC staining

DAB IHC staining was performed on the entire cohort. 4 μm-thick FFPE sections of NC, LGCA and HGCA tissue samples were stained for iPSC markers OCT4, SOX2, NANOG, KLF4 and c-MYC. Positive human control tissues included in each run to validate the staining were seminoma for OCT4 and NANOG, normal skin for SOX2, normal breast tissue for KLF4 and prostatic tissue for c-MYC. Each IHC staining procedure also included a matched isotype antibody as a negative control. Protocols were performed as previously described [29].

Staining was carried out on the Leica BOND^™^ RX Auto-stainer using primary antibodies for OCT4 (1:30; cat#MRQ-10, Cell Marque, Rocklin, CA, USA), SOX2 (1:200; cat#ab97959, Abcam, Cambridge, MA, USA), NANOG (1:200; cat#EP225, Cell Marque), KLF4 (1:200; cat#NBP2-24749SS, Novus Biologicals LLC, Littleton, CO, USA) and c-MYC (1:1000; cat#ab32, Abcam).

### IF IHC staining

Protein localization was performed on three LGCA and three HGCA and their patient-matched normal colon samples by dual IF IHC staining, carried out on the Leica BOND^™^ RX Auto-stainer. Secondary antibodies used were Vectafluor Excel goat anti-mouse 488 (ready-to-use; cat#DK2488, Vector Laboratories, Burlingame, CA, USA) and Alexa Fluor donkey anti-rabbit 594 (1:500; cat#ab150076, Life Technologies, Carlsbad, CA, USA). All stained slides were mounted as previously described [29]. Negative controls were performed using matched isotype controls for both mouse (ready-to-use; cat#IR750, Dako, Copenhagen, Denmark) and rabbit (ready-to-use; cat#IR600, Dako).

### ISH

ISH staining was performed on 4 μm-thick FFPE sections of six LGCA and six HGCA tissue samples and their patient-matched NC tissue samples. This was carried out on the Leica BOND^™^ RX Auto-stainer using probes for OCT4 (NM_002701), SOX2 (NM_003106), NANOG (NM_024865), KLF4 (NM_004235) and c-MYC (NM_002467), using the ViewRNA eZ Detection Kit to detect the presence of mRNA (Affymetrix, Santa Clara, CA, USA). Positive controls were human seminoma for OCT4, NANOG and KLF4, normal skin for SOX2, and normal colon tissue for c-MYC. To determine specificity of the probes, negative controls were run using a probe for *Bacillus* (NM_L38424).

### Image capture and analysis

DAB IHC and ISH images were captured using an Olympus BX53 light microscope fitted with an Olympus SC100 digital camera and CellSens 2.0 software (Olympus, Tokyo, Japan). Images were used for manual cell counting using ImageJ software (National Institutes of Health, Bethesda, MD, USA). Six images were captured per stained slide. When capturing images, areas with muscle and blood vessels were avoided. All positively and negatively stained cells within these images were counted and identified as either epithelial (crypt) or stromal cells. Cell were counted as positive if they had any level of staining (weak, moderate or strong) in the nucleus and/or cytoplasm. IF IHC-stained slides were visualized and imaged using an Olympus FV1200 biological confocal laser-scanning microscope (Olympus) and processed using CellSens 2.0 software (Olympus).

### RNA extraction

RNA was extracted from the same cohorts of six LGCA and six HGCA tissue samples and their patient-matched NC tissues, using a QIAcube (Qiagen) as previously described [30].

### RT-qPCR

RT-qPCR reactions were run on the Rotor-Gene Q (Qiagen), as previously described [30] using Taqman primers (Thermo Fisher) for OCT4 (Hs00999632_g1; 77kb), SOX2 (Hs01053049_s1; 91kb), NANOG (Hs04399610_g1; 101kb), KLF4 (Hs00358836_m1; 110kb) and c-MYC (Hs00153408_m1; 107kb).

### Statistical analysis

Statistical analysis was carried out using SPSS V22. Protein expression of iPSC markers in the stroma and the crypt were compared using a *t*-test for both normal and tumor samples. Statistical significance was defined as a *p*<0.05.

The differences between LGCA and HGCA tissue samples were calculated using Analysis of Variance (ANOVA). A discriminant function analysis was also performed using the four sets of data which had the highest correlation for either LGCA or HGCA tumors. This produced a canonical correlation value and Wilkes Lambda variance value, representing the level of confidence for which these four pieces of data taken from any given specimen is able to be used to predict the grade of the tumor. mRNA levels were compared between normal stroma and tumor stroma, and between normal epithelium and tumor epithelium, using a *t*-test to determine statistical significance.

## Results

### DAB IHC staining

EPCAM was used to distinguish between epithelial cells and stromal cells (S1 Fig). It was found that EPCAM expression was restricted to epithelial cells in all NC, LGCA and HGCA tissues.

**Fig 1:**
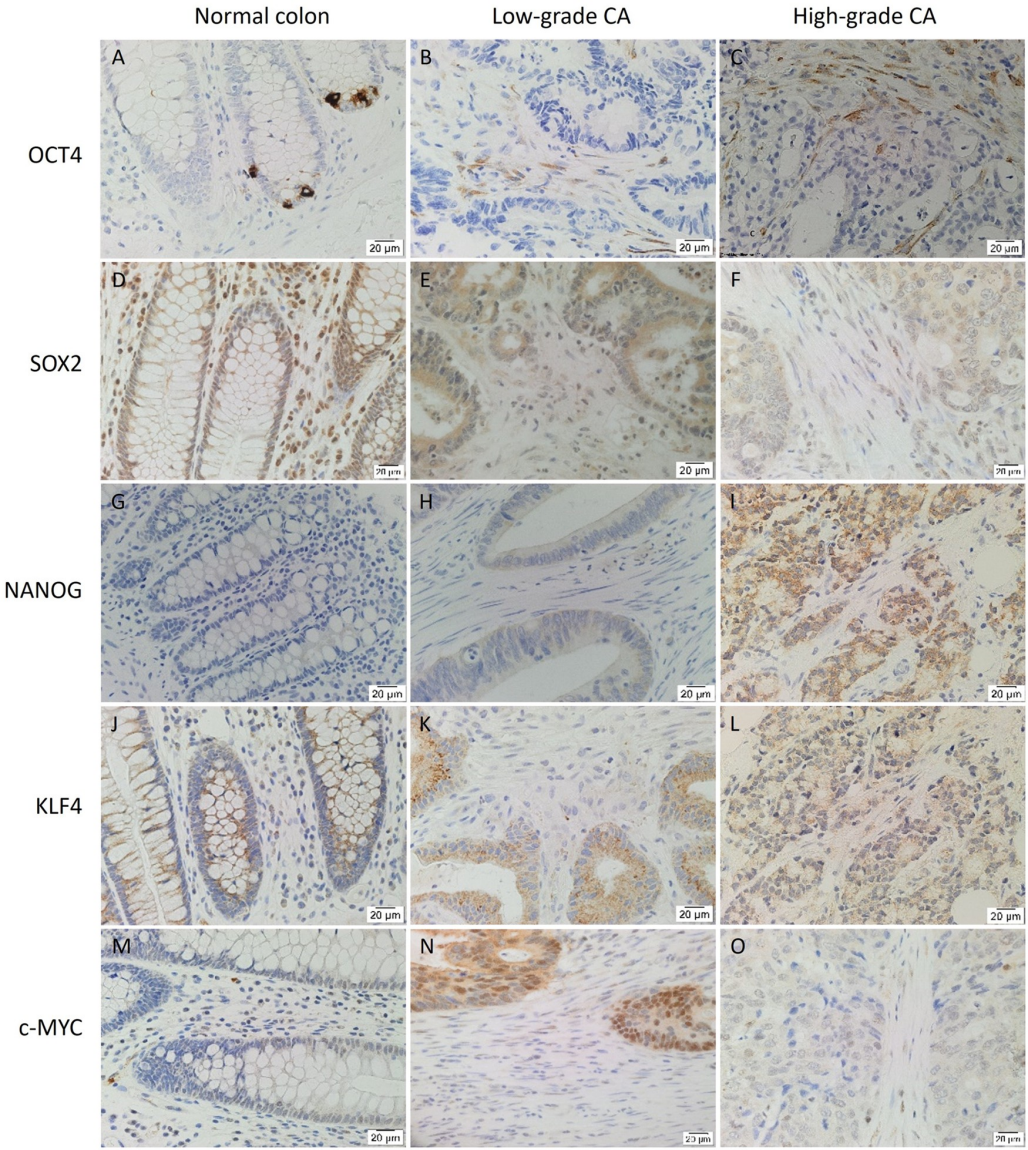
DAB IHC staining. Representative 3,3-diaminobenzidine immunohistochemical-stained images showing protein expression of induced-pluripotent stem cell markers OCT4 (A-C, brown), SOX2 (D-F, brown), NANOG (G-I, brown), KLF4 (J-L, brown) and c-MYC (M-O, brown) in normal colon (A,D,G,J,M), low-grade (B,F,H,K,N) and high-grade (C,F,I,L,O) colon adenocarcinoma tissue samples. Nuclei were counter-stained with hematoxylin (A-O, blue). Original magnification: 400x.

OCT4 (Fig 1A–1C, brown) was detected in the nucleus of 2.5% of NC epithelial cells, likely to be the normal intestinal stem cells (A). However, it was found in the cytoplasm of 25% of stromal cells in LGCA (B) and 30% of stromal cells in HGCA (C) with little or no presence in the epithelium (0.7%). SOX2 (Fig 1D–1F, brown) was expressed in the cytoplasm of epithelial and stromal cells in NC, LGCA and HGCA tissue samples. Overall, NC (D) samples stained more strongly than LGCA (E) and HGCA (F) tissue samples. SOX2 was abundant in the nuclei of NC epithelium. NANOG (Fig 1G–1I, brown) was not detected in NC (G) but was present in HGCA (75% of cases, weak-to-moderate; H) and LGCA (40% of cases, weak; I) tissue samples. c-MYC (Fig 1J–1L, brown) was observed in the nuclei and cytoplasm of NC (J), LGCA (K) and HGCA (L) epithelium. KLF4 (Fig 1M–1O, brown) showed perinuclear expression in NC epithelial cell cytoplasm (M). HGCA epithelium (N) exhibited more nuclear staining than LGCA (O) and NC (M) epithelium. KLF4 expression was seen in 22% of NC stromal cells of LGCA samples, and 44% of NC stromal cells of HGCA samples. Its expression was lower in LGCA than the NC samples with 14.4% of LGCA stromal cells staining positively, while 47.9% of stromal cells in HGCA samples expressed KLF4. Positive and negative controls are shown in S2 Fig, and cell counting data is displayed in S1 Table.

**Fig 2:**
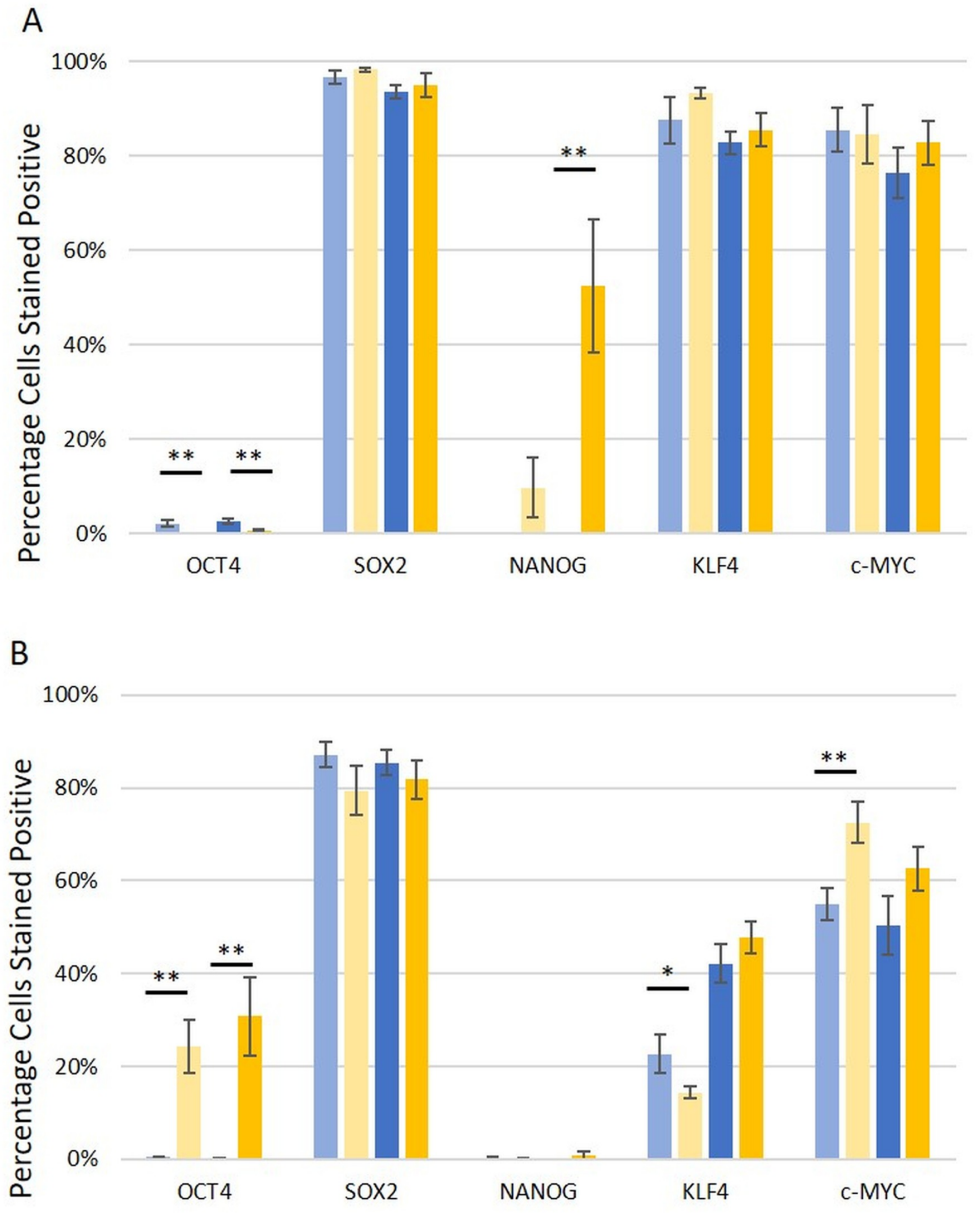
DAB IHC data. Percentage of cell population stained positively for induced-pluripotent stem cell markers OCT4, SOX2, NANOG, KLF4 and c-MYC by 3,3-diaminobenzidine immunohistochemical staining, for the epithelium (A) and the stroma (B). Normal colon samples from patients with low-grade colon adenocarcinoma (LGCA; pale blue, n = 9) are displayed separately to normal colon samples from patients with high-grade colon adenocarcinoma (HGCA; dark blue, n = 8). LGCA samples are shown in pale yellow (n = 10), and HGCA samples are shown in dark yellow (n = 8). Statistical significance with a *p*-value between 0.05 and 0.01 is shown by *, and that for <0.01 is represented by **. Error bars show standard error.

Two CSC subpopulations were identified by DAB IHC staining (Fig 2): one within the CA epithelium, with 9.7% of LGCA and 52.4% of HGCA epithelial cells expressing NANOG (Fig 2A); and the other within the CA stroma, with OCT4 being expressed by 24.3% of LGCA and 30.8% of HGCA stromal cells (Fig 2B). Discriminant value analysis revealed that all LGCA and HGCA tissue samples could be graded with 100% accuracy based on stromal expression of KLF4 in NC (*p* = 0.020) and the tumors (*p* = 0.034), and OCT4 (*p* = 0.001) and NANOG (*p* = 0.026) in CA epithelium (canonical correlation = 0.981; Wilkes Lambda = 0.037).

### IF IHC staining

IF IHC staining expanded on DAB IHC data by revealing localization of two iPSC markers simultaneously, as well as being a more sensitive detection method.

**Fig 3:**
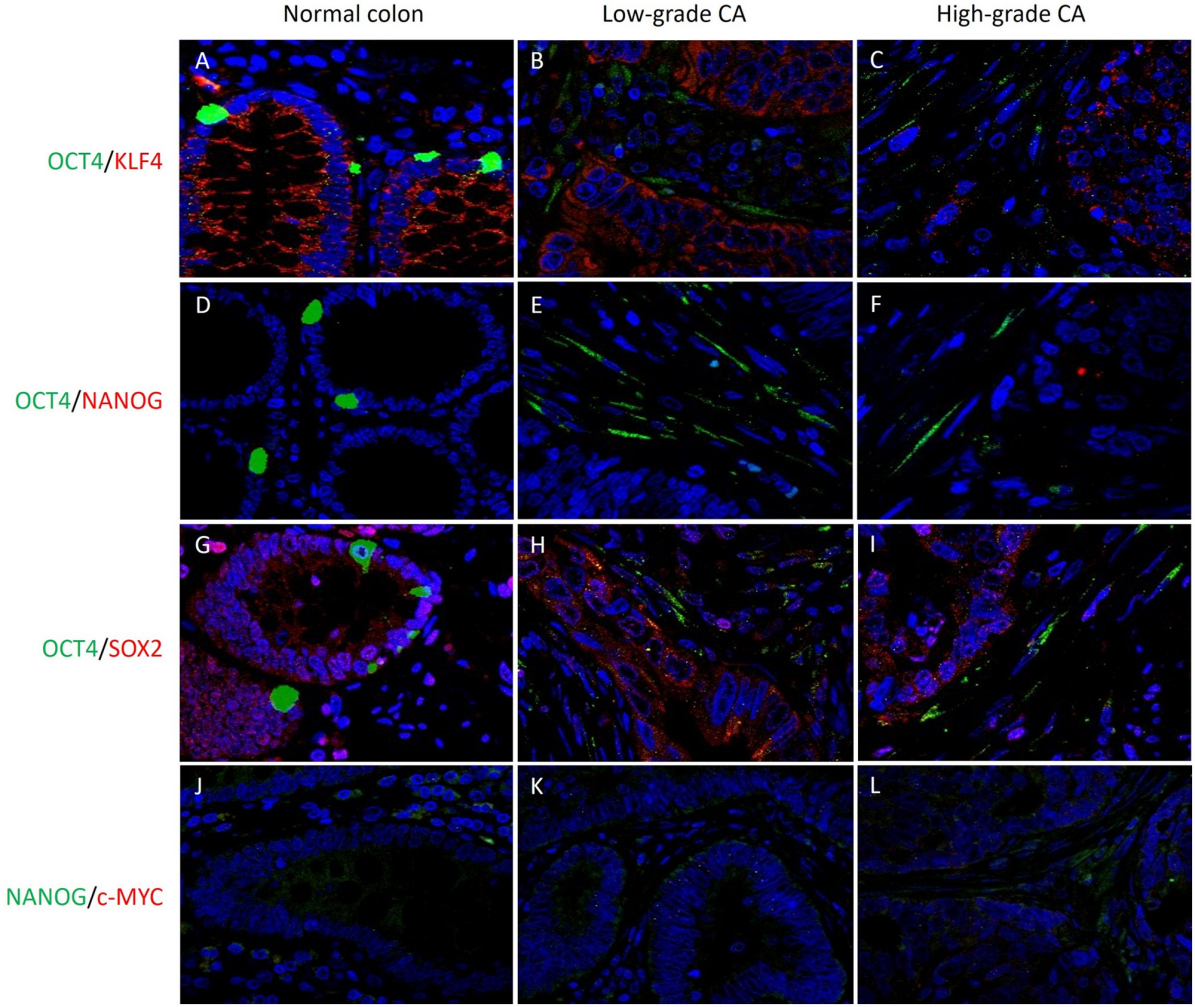
IF IHC staining. Representative immunofluorescence immunohistochemical-stained images showing protein expression of induced-pluripotent stem cell markers OCT4 (A-J, green), KLF4 (A-C, red), NANOG (D-G, H-J, red), SOX2 (H-J, red), and c-MYC (H-J, green) in normal colon (A,D,E,H), low-grade (B,E,F,I) and high-grade (C,F,G,J) colon adenocarcinoma tissue samples. Cell nuclei were counter-stained with 4’, 6’-diamidino-2-phenylindole (A-L, blue). Original magnification: 400x.

OCT4 (Fig 3A–3I, green) was expressed in the nucleus of few epithelial cells in NC (Fig 3A, 3D and 3G) and the cytoplasm of cells in the stroma of LGCA (Fig 3B, 3E and 3H) and HGCA (Fig 3C, 3F and 3I) tissue samples. KLF4 (Fig 3A–3C, red) stained positively in the cytoplasm of epithelial cells in NC (Fig 3A), LGCA (Fig 3B) and HGCA (Fig 3C), and some stromal cells in LGCA (Fig 3B, red) and HGCA (Fig 3C) samples. Epithelial cells in NC (Fig 3A) but not the stromal cells in LGCA (Fig 3B) or HGCA (Fig 3C) samples co-expressed OCT4 and KLF4 in their cytoplasm. NANOG (Fig 3D–3F and 3J–3L, red) was absent in NC (Fig 3D and 3J) but was seen in the cytoplasm of epithelial cells in LGCA (Fig 3E and 3K) and HGCA (Fig 3F and 3L) samples. SOX2 (Fig 3G–3I, red) was widely expressed in the epithelial cells and some stromal cells in both NC (Fig 3A), LGCA (Fig 3H) and HGCA (Fig 3I) tissue samples. SOX2 and OCT4 were co-expressed in epithelial cells in NC (Fig 3G) and stromal cells in LGCA (Fig 3H) and HGCA (Fig 3I). Cytoplasmic and nuclear staining of c-MYC (Fig 3J–3L, green) was weak in NC (Fig 3J), LGCA (Fig 3K) and HGCA (Fig 3L) epithelial and stromal cells. Stromal cells co-expressing OCT4 and SOX2 and those that stained positively for c-MYC had the same morphology and were assumed to be the same cell type. c-MYC and NANOG were co-expressed in HGCA epithelial cells (Fig 3L). From the above data we inferred that there was an epithelial subpopulation co-expressing NANOG, SOX2 and KLF4, and a stromal subpopulation co-expressing OCT4, SOX2 and c-MYC. Split images for Fig 3 are shown in S3 Fig.

### RT-qPCR

**Fig 4:**
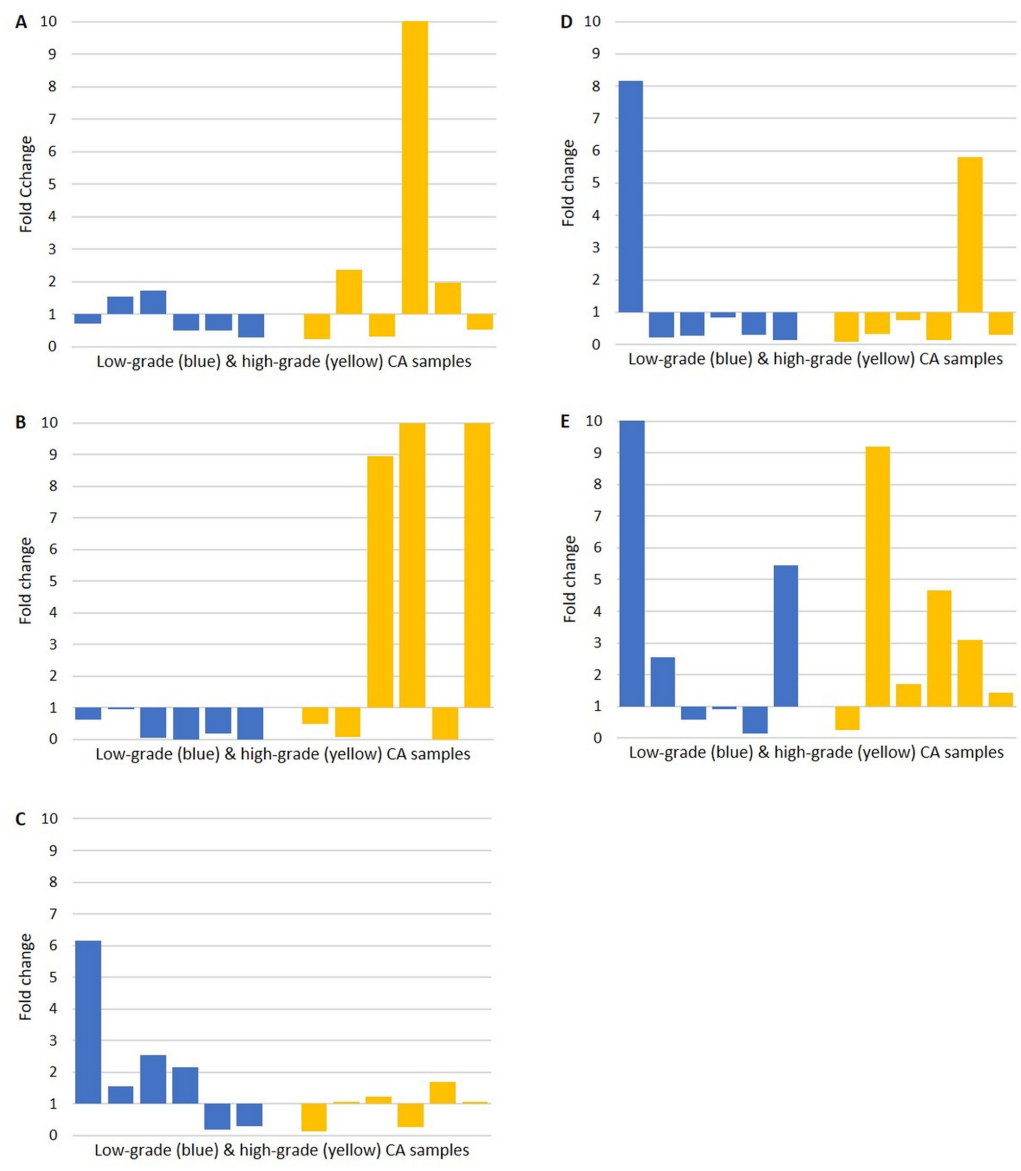
RT-qPCR. mRNA expression of induced-pluripotent stem cell markers *OCT4* (A), *SOX2* (B), *NANOG* (C), *KLF4* (D) and *c-MYC* (E) detected by RT-qPCR. Data displayed as the fold-change of gene expression in tumor samples relative to their patient-matched normal colon sample (Y-axis). A cohort of six LGCA tissue samples are shown in blue, and a cohort of six HGCA tissue samples are shown in yellow (X-axis).

RT-qPCR demonstrated mRNA expression of all five iPSC markers in both the NC, LGCA and HGCA tissue samples (Fig 4). *SOX2* mRNA was below the detection threshold in three NC tissue samples, and *OCT4* was not detected in one NC tissue sample. *NANOG*, *KLF4* and *c-MYC* mRNA was detected in all 12 NC tissue samples. All LGCA and HGCA tissue samples expressed mRNA for *OCT4*, *NANOG*, *KLF4* and *c-MYC*. One LGCA and one HGCA tissue sample did not reach the detection threshold for *SOX2*. ΔCT and fold-change data is displayed in S2 Table.

### ISH

**Fig 5:**
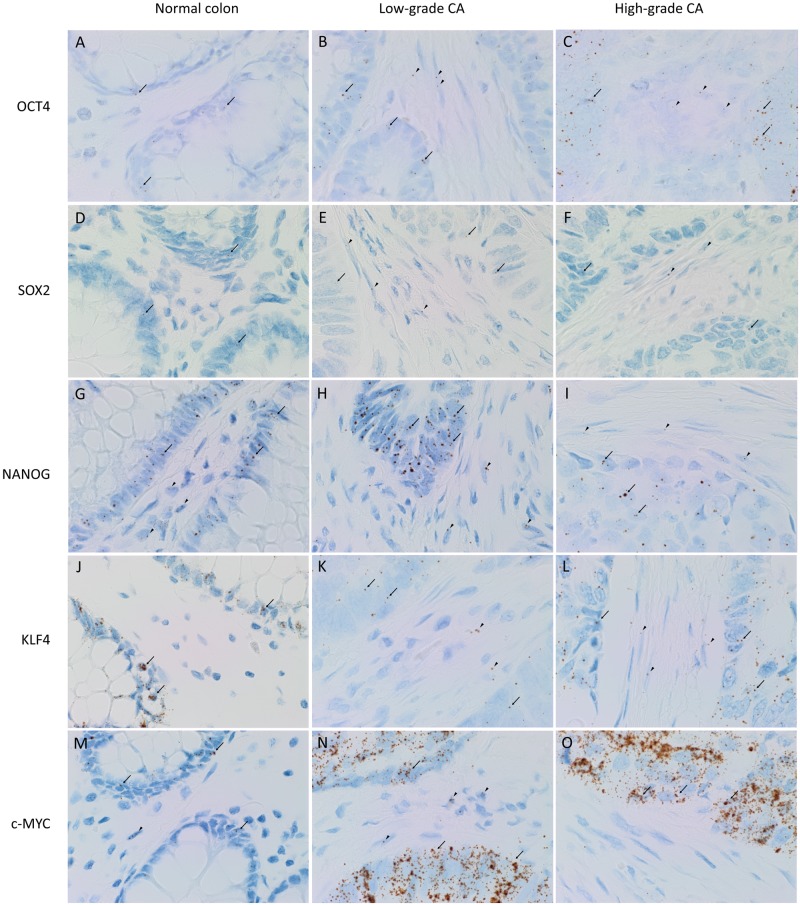
*In-situ* hybridization. Representative images of *in-situ* hybridization, showing mRNA expression of iPSC genes *OCT4* (A-C, brown), *SOX2* (D-F, brown), *NANOG* (G-I, brown), *KLF4* (J-L, brown) and *c-MYC* (M-O, brown) in the epithelial cells (*arrows*) and stromal cells (*arrowheads*) in normal colon (A,D,G,J,M), low-grade (B,E,H,K,N) and high-grade (C,F,I,L,O) colon adenocarcinoma tissue samples. Nuclei were counter-stained with hematoxylin (blue). Original magnification: 1000x.

ISH demonstrated the presence of mRNA for *OCT4* (Fig 5A–5C, brown), *SOX2* (Fig 5D–5F, brown), *NANOG* (Fig 5G–5I, brown), *KLF4* (Fig 5J–5L, brown) and c*-MYC* (Fig 5M–5O, brown) in NC (Fig 5A, 5D, 5G, 5J and 5M), LGCA (Fig 5B, 5E, 5H, 5K and 5N) and HGCA (Fig 5C, 5F, 5I, 5L and 5O) tissue samples. Positive and negative controls are shown in S4 Fig, and cell counting data is displayed in S3 Table.

**Fig 6:**
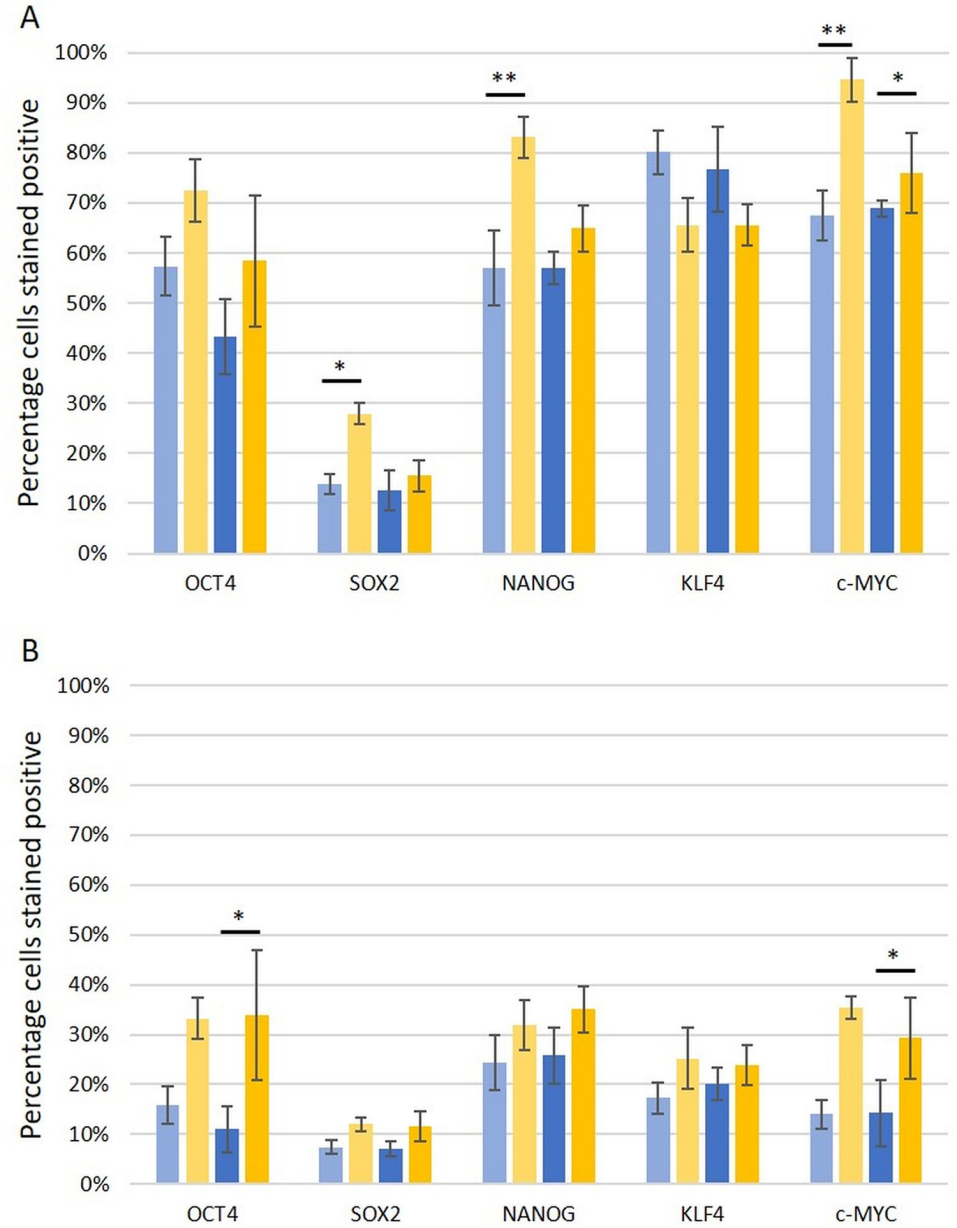
*In-situ* hybridization data. *In-situ* hybridization analysis showing the percentage of the cell population expressing mRNA for iPSC genes in the epithelium (A) and the stroma (B). Normal colon samples from patients with low-grade colon adenocarcinoma (LGCA) are represented in pale blue, normal colon samples from high-grade colon adenocarcinoma (HGCA) patients in dark blue, LGCA samples in pale yellow, and HGCA samples in dark yellow. Statistical significance with a *p*-value between 0.05 and 0.01 is shown by *, and that for <0.01 is represented by **. Error bars show standard error.

ISH cell counting demonstrated *OCT4*, *SOX2*, *NANOG* and *c-MYC* had higher mRNA levels in CA epithelium and stroma when compared to NC (Fig 6). Conversely, *KLF4* was more highly expressed in NC epithelium than that of CA. All differences between CA and their patient-matched NC tissue samples showed statistical significance (*p*<0.05).

## Discussion

This study investigated the transcriptional and translational expression of *OCT4*, *SOX2*, *NANOG*, *KLF4* and *c-MYC* to identify their presence in CSC subpopulations within CA.

RT-qPCR and ISH data for *SOX2* corroborated with each other, with RT-qPCR failing to detect *SOX2* in three NC samples and two CA samples, and ISH showing *SOX2* to be the least abundant in terms of the number of cells containing mRNA. However, SOX2 was one of the most abundant markers at the protein level. Other studies have also shown an abundance of SOX2 protein in both the nuclei and cytoplasm of CRC tumor cells [11, 19]. Furthermore, ISH showed abundance of c-MYC mRNA but DAB IHC staining showed weak protein staining, which may be due to the concentration of primary antibody used for DAB IHC staining.

KLF4 has been previously studied in CRC and shown to be associated with epithelial-to-mesenchymal transition (EMT), cell migration and metastasis [16]. However, studies on the role of KLF4 in cancer often yield conflicting results [31]. In NC, KLF4 helps direct epithelial progenitor cells down the goblet cell lineage, the most abundant epithelial cell type in colonic crypts [26]. As the grade of CA increases, the tumors are less differentiated, and this may explain the observation of decreased KLF4 in both LGCA and HGCA tumors, relative to NC. Our finding of higher KLF4 and OCT4 protein expression in the stroma of HGCA may reflect the migration of cancer cells by EMT, away from the epithelium, which has been postulated as a major factor in CRC progression [32]. Furthermore, when applying the concept of a stem cell hierarchy in cancer [6–8], we propose that cells at different levels of this hierarchy will express different combinations of these markers. For instance, OCT4 is known to be expressed by primitive stem cells such as ESCs [16, 33], whereas KLF4 is associated with a more differentiated phenotype [26, 27].

In this study, IF IHC staining identified two distinct CSC subpopulations: a NANOG^+^/OCT4^-^ subpopulation localized to the epithelium, and an OCT4^+^/NANOG^-^ subpopulation within the stroma. The stromal OCT4^+^ subpopulation did not co-express NANOG or KLF4, but some stromal cells co-expressed OCT4 and SOX2. Similarly, in the epithelium, SOX2 and KLF4 staining was widespread but comparatively few of these cells were NANOG^+^. Based on the staining patterns, of these markers we infer the presence of two predominant CSC subpopulations in CA: an OCT4^+^/SOX2^+^/c-MYC^+^ subpopulation within the stroma and a NANOG^+^/SOX2^+^/KLF4^+^ subpopulation within the epithelium.

The literature correlating OCT4 and SOX2 expression with EMT and metastasis provides evidence supporting a stromal subpopulation expressing these markers that migrates away from the tumor [20, 31, 34]. Furthermore, NANOG is associated with maintenance of the stem-like phenotype of CSCs within the tumor, consistent with our observation of NANOG expression by some epithelial cells within the tumor [35]. KLF4 and c-MYC are associated with proliferation and differentiation and it is therefore not unexpected that these two markers were co-expressed by cells within CA [24, 27, 36].

Some stromal cells within CA that stained positively for OCT4 and c-MYC did not express KLF4 and SOX2. Some of these OCT4^+^ stromal cells may be cancer-associated fibroblasts recruited by the tumor and induced to express OCT4 [37]. Alternatively, they may represent a CSC subpopulation that expresses stem cell markers other than the iPSC-related genes investigated in this study.

The patient-matched ‘normal colon’ samples used as a control may not represent true normal colon. Other limitations of this study include the lack of functional *in vitro* and *in vivo* investigations which will be the focus of future work.

The significance of OCT4, NANOG and KLF4 expression was seen in the discriminant values analysis. By considering the expression of stromal KLF4 and epithelial OCT4 and NANOG, all 18 CA cases could be accurately graded. This is demonstrated to be robust, with a canonical correlation of 0.981 representing a high degree of statistical significance, and a Wilkes Lambda value of 0.037 showing that 96.3% of the variance between cases can be explained by these data.

Once validated, using the localization and expression levels of novel combinations of iPSC markers may provide a valuable tool to help guide patient management by further stratifying tumor grade, identifying cases with higher potential for metastasis or relapse, or tracking response to therapy.

## Supporting information

S1 Fig EpCAM DAB IHC staining. Representative 3,3-diaminobenzidine immunohistochemical images showing protein expression of EPCAM (brown) in normal colon (A), low-grade colon adenocarcinoma (B&C), negative control (D), and high-grade colon adenocarcinoma (E&F). In all normal and tumor samples, EPCAM was expressed only by the epithelial cells and not by stromal cells. Nuclei were counter-stained with hematoxylin (A-O, blue). Original magnification: 400x.
(TIF)

S2 Fig DAB IHC controls. Representative images of 3,3 diaminobenzidine immunohistochemical staining of human positive control tissues demonstrating the expected staining patterns on seminoma for OCT4 (A, brown) and NANOG (B, brown), skin for SOX2 (C, brown), normal breast tissue for KLF4 (D, brown) and prostatic tissue for c-MYC (E, brown). A section of colon adenocarcinoma probed with a matched anti-mouse isotype control and primary antibodies (F) confirmed the specificity of the secondary antibodies. Nuclei were counter-stained with hematoxylin (B-F, blue). Original magnification: 400X.
(TIF)

S3 Fig IF IHC controls. Individual stains of immunofluorescence immunohistochemical staining of normal colon (A,B,G,H,M,N,S,T), low-grade (C,D,I,J,O,P,U,V), and high-grade (E,F,K,L,Q,R,W,X) colon adenocarcinoma samples shown in Fig 3. Sections were co-stained for OCT4 (B,D,F,H,J,L,N,P,R, green) with KLF4 (A,C,E, red), NANOG (G,I,K, red) and SOX2 (M,O,Q, red); and c-MYC (T,V,X, green) with NANOG (S,U,W, red). Cell nuclei were counter-stained with 4’6-diamino-2-phenylinodole (A-X). Scale bars: 20μm.
(TIF)

S4 Fig ISH controls. *In-situ* hybridization positive human control tissues for OCT4 (A, brown), NANOG (B, brown) and KLF4 (C, brown) on seminoma; SOX2 (D, brown) on normal skin, and c-MYC (E, brown) on normal colon. Negative control (F) performed on sections of colon adenocarcinoma tissue sample confirms specificity of secondary antibody. Original magnification: 1000x.
(TIF)

S1 Table DAB IHC cell counting data. Data showing the percentage of cells with any protein expression of each induced-pluripotent stem cell (iPSC) marker (weak, moderate or strong) by cells in the epithelium and those in the stoma with the standard error values in brackets. LGCA, low-grade colon adenocarcinoma tissue samples; HGCA, high-grade colon adenocarcinoma tissue samples. NCLG, normal colon tissue from patients with LGCA; NCHG, normal colon tissue from patients with HGCA. Significance values for comparisons between LGCA and HGCA tissue samples and their patient-matched normal colon tissues, for cells in the epithelium and those in the stroma: a p-value between 0.05 and 0.01 is shown by *, and <0.01 represented by **.
(PDF)

S2 Table RT-qPCR data. RT-qPCR data showing expression of induced-pluripotent stem cell (iPSC) markers *OCT4*, *SOX2*, *NANOG*, *KLF4* and *c-MYC*. ΔCT values calculated by comparing the gene of interest to housekeeper *GAPDH*, and ΔΔCT values by comparing high-grade (HG) and low-grade (LG) tumors to their patient-matched normal colon samples. ΔΔCT values used to calculate fold changes using the equation 2^(-ΔΔCT).
(PDF)

S3 Table ISH cell counting data. Data showing the percentage of cells with mRNA expression of each induced-pluripotent stem cell (iPSC) marker in the epithelium and in the stoma, with the standard error values in brackets. LGCA, low-grade colon adenocarcinoma tissue samples; HGCA, high-grade colon adenocarcinoma tissue samples. NCLG, normal colon tissue from patients with LGCA; NCHG, normal colon tissue from patients with HGCA. Significance values for comparisons between LGCA and HGCA tissue samples and their patient-matched normal colon tissues, for cells in the epithelium and those in the stroma: a p-value between 0.05 and 0.01 is shown by *, and <0.01 represented by **.
(PDF)

S1 Datasets Raw data. Raw data collected and analyzed during this study is provided, including anonymous patient data, DAB IHC cell counting data, discriminant function analysis output, *in-situ* hybridization cell counting data, raw RT-qPCR CT values, and cell counting statistics.
(ZIP)
