# Supplementary figures and images for: Cancer stem cell subpopulations in primary colon adenocarcinoma
Source: PLoS One. 2019 Sep 6;14(9):e0221963. doi: 10.1371/journal.pone.0221963 (PMC6730900; doi:10.1371/journal.pone.0221963)

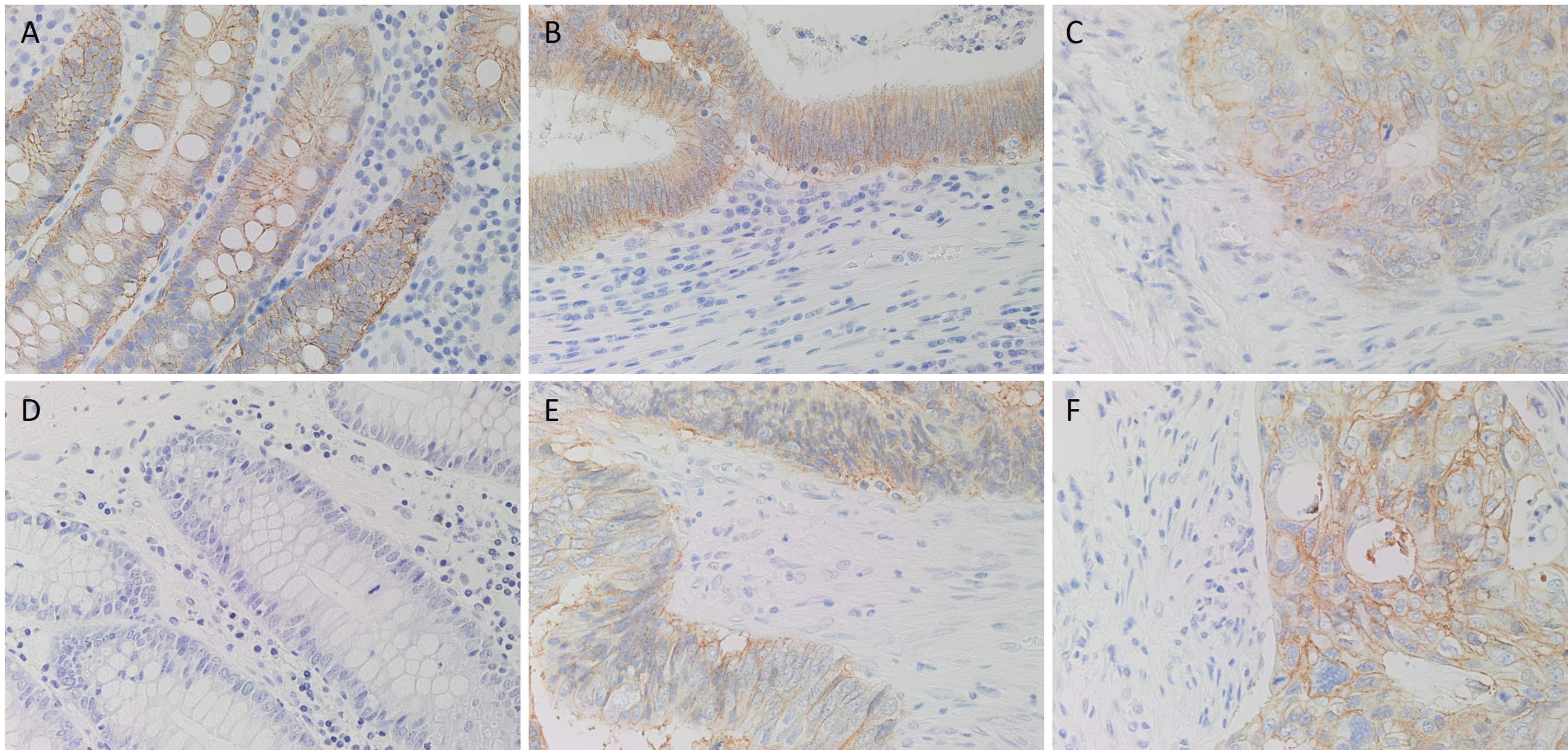

Supplement: S1 Fig — Representative 3,3-diaminobenzidine immunohistochemical images showing protein expression of EPCAM (brown) in normal colon (A), low-grade colon adenocarcinoma (B&C), negative control (D), and high-grade colon adenocarcinoma (E&F). In all normal and tumor samples, EPCAM was expressed only by the epithelial cells and not by stromal cells. Nuclei were counter-stained with hematoxylin (A-O, blue). Original magnification: 400x. (TIF) [file pone.0221963.s001.tif]

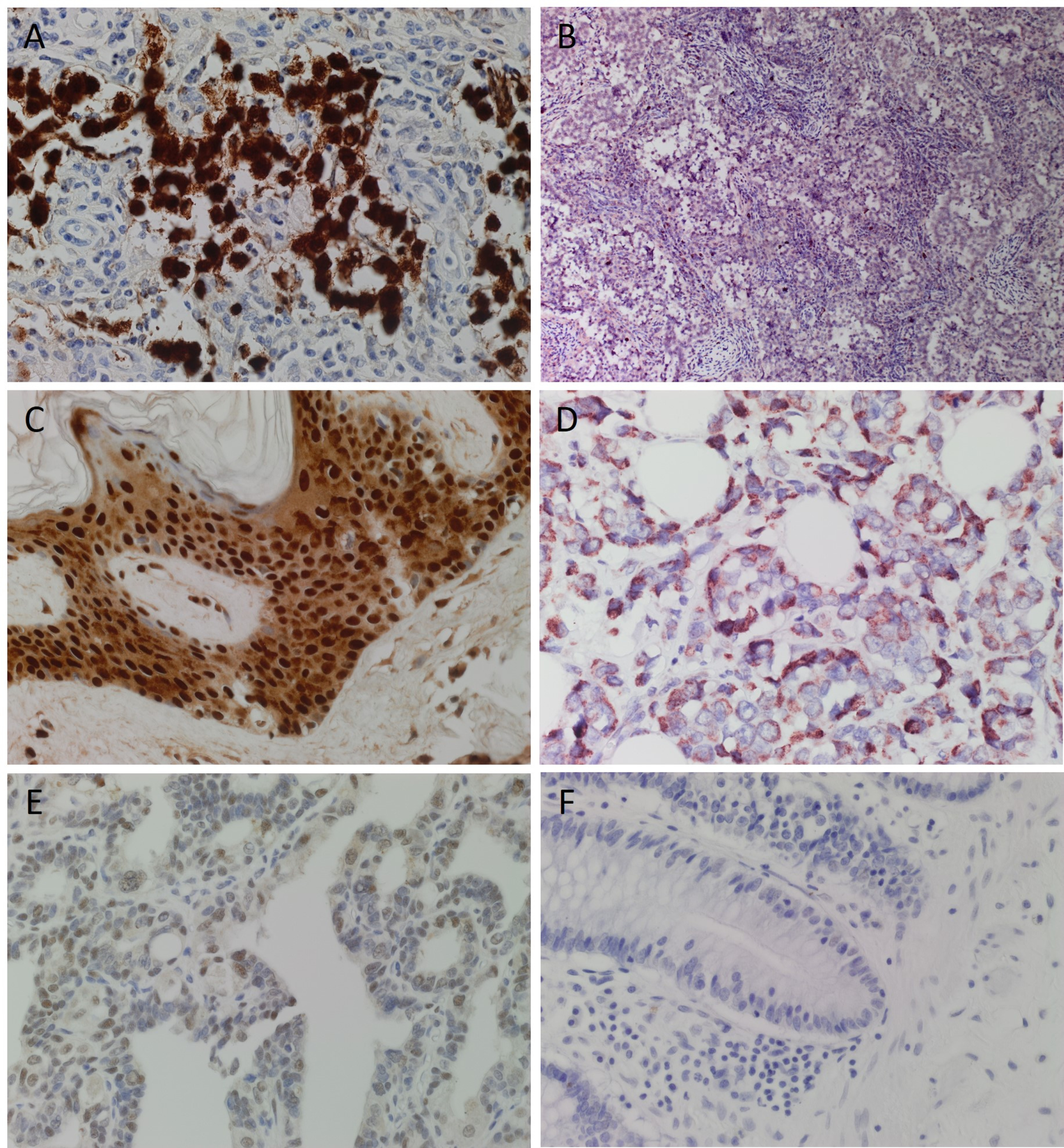

Supplement: S2 Fig — Representative images of 3,3 diaminobenzidine immunohistochemical staining of human positive control tissues demonstrating the expected staining patterns on seminoma for OCT4 (A, brown) and NANOG (B, brown), skin for SOX2 (C, brown), normal breast tissue for KLF4 (D, brown) and prostatic tissue for c-MYC (E, brown). A section of colon adenocarcinoma probed with a matched anti-mouse isotype control and primary antibodies (F) confirmed the specificity of the secondary antibodies. Nuclei were counter-stained with hematoxylin (B-F, blue). Original magnification: 400X. (TIF) [file pone.0221963.s002.tif]

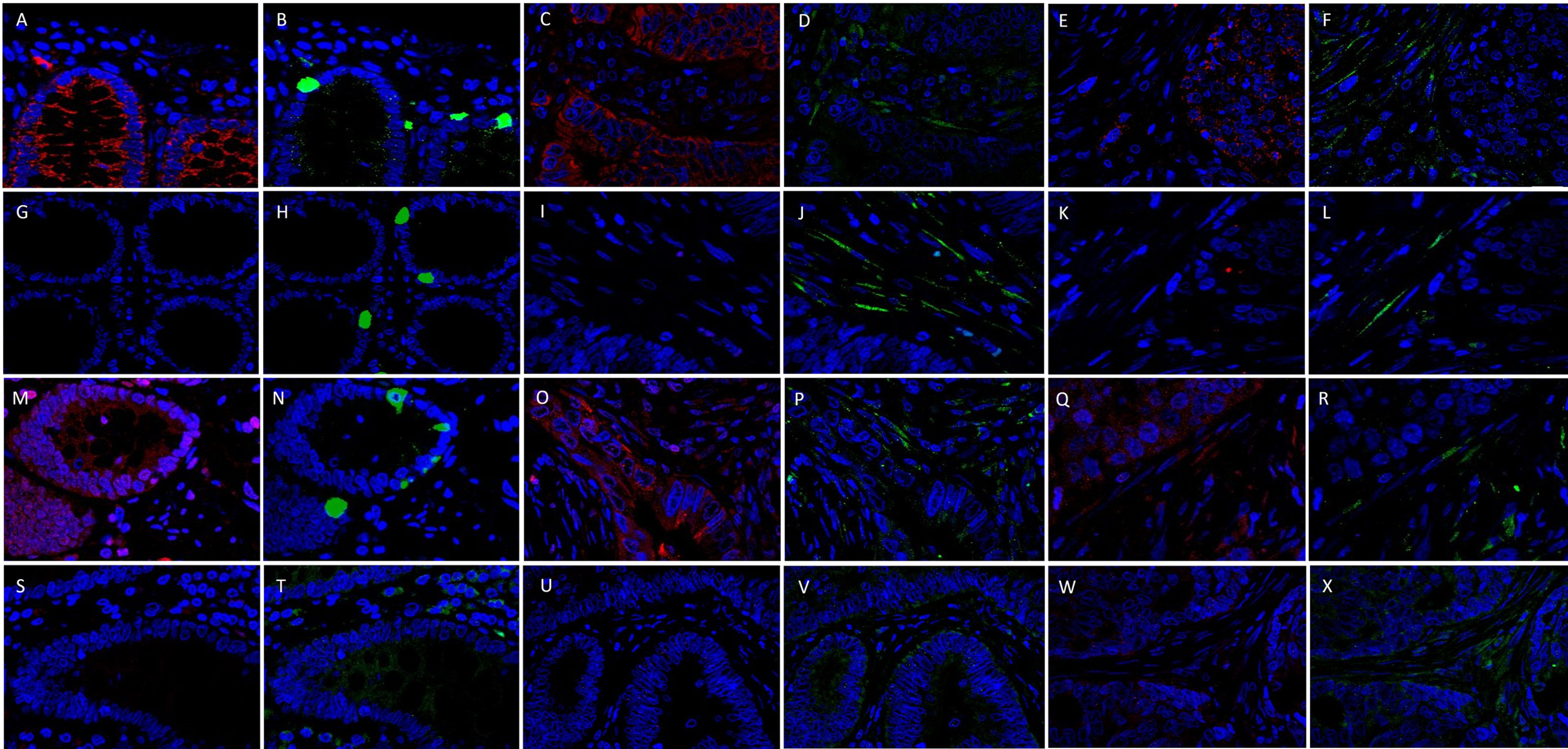

Supplement: S3 Fig — Individual stains of immunofluorescence immunohistochemical staining of normal colon (A,B,G,H,M,N,S,T), low-grade (C,D,I,J,O,P,U,V), and high-grade (E,F,K,L,Q,R,W,X) colon adenocarcinoma samples shown in Fig 3. Sections were co-stained for OCT4 (B,D,F,H,J,L,N,P,R, green) with KLF4 (A,C,E, red), NANOG (G,I,K, red) and SOX2 (M,O,Q, red); and c-MYC (T,V,X, green) with NANOG (S,U,W, red). Cell nuclei were counter-stained with 4’6-diamino-2-phenylinodole (A-X). Scale bars: 20μm. (TIF) [file pone.0221963.s003.tif]

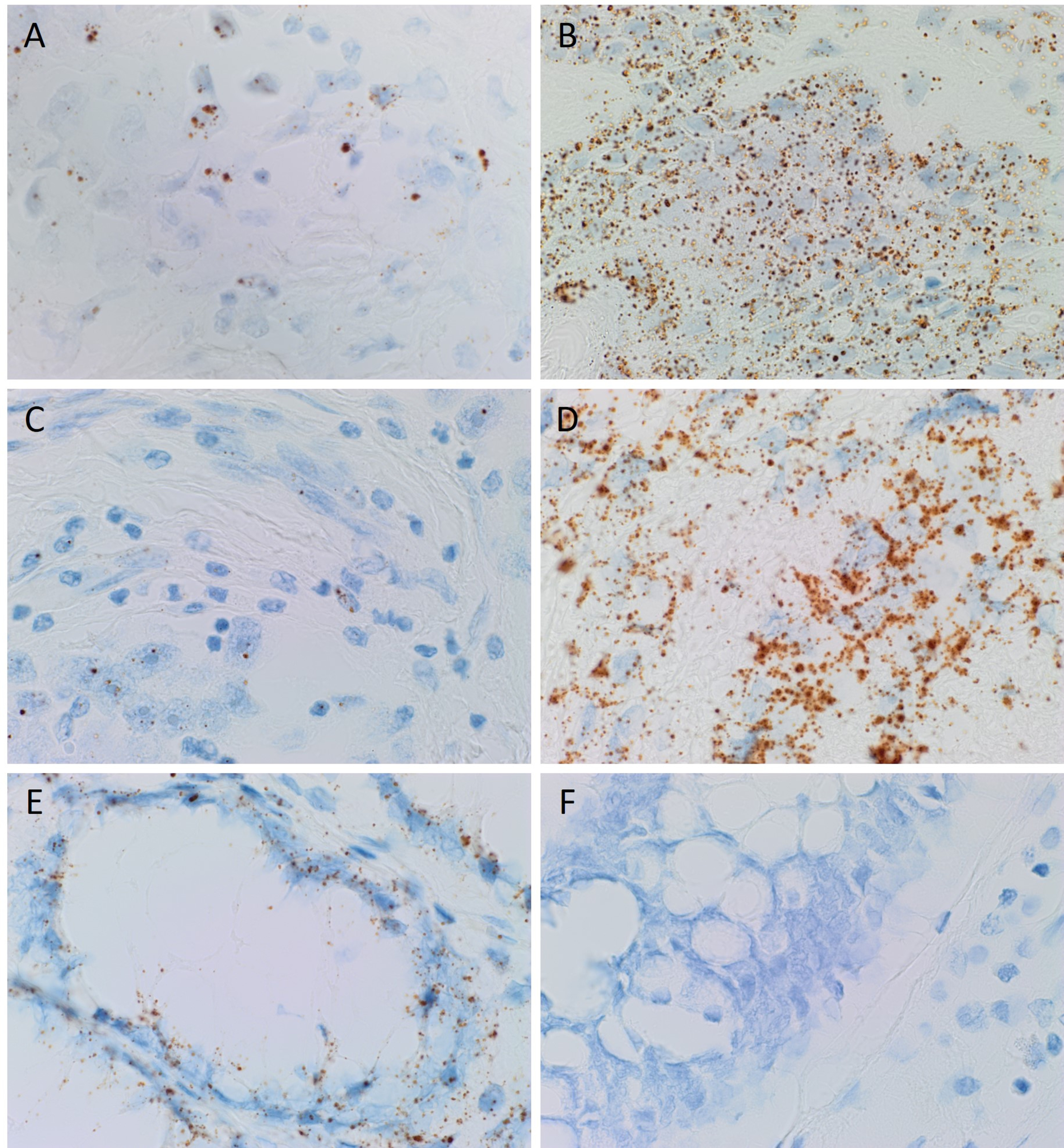

Supplement: S4 Fig — In-situ hybridization positive human control tissues for OCT4 (A, brown), NANOG (B, brown) and KLF4 (C, brown) on seminoma; SOX2 (D, brown) on normal skin, and c-MYC (E, brown) on normal colon. Negative control (F) performed on sections of colon adenocarcinoma tissue sample confirms specificity of secondary antibody. Original magnification: 1000x. (TIF) [file pone.0221963.s004.tif]
