# Supplementary material for: Cancer stem cell subpopulations in primary colon adenocarcinoma
Source: PLoS One. 2019 Sep 6;14(9):e0221963. doi: 10.1371/journal.pone.0221963 (PMC6730900; doi:10.1371/journal.pone.0221963)
Supplement: S1 Table — Data showing the percentage of cells with any protein expression of each induced-pluripotent stem cell (iPSC) marker (weak, moderate or strong) by cells in the epithelium and those in the stoma with the standard error values in brackets. LGCA, low-grade colon adenocarcinoma tissue samples; HGCA, high-grade colon adenocarcinoma tissue samples. NCLG, normal colon tissue from patients with LGCA; NCHG, normal colon tissue from patients with HGCA. Significance values for comparisons between LGCA and HGCA tissue samples and their patient-matched normal colon tissues, for cells in the epithelium and those in the stroma: a p-value between 0.05 and 0.01 is shown by *, and <0.01 represented by **. (PDF) [file pone.0221963.s005.pdf]

S1 Table: 3,3-Diaminobenzidine immunohistochemical staining cell counting data

		iPSC Markers				
		OCT4	SOX2	NANOG	KLF4	c-MYC
Epithelium	NCLG	2.19% (0.0073)	96.70% (0.0134)	0.02% (0.0003)	86.87% (0.0482)	84.96% (0.0468)
	LGCA	0.03% (0.0002)	98.42% (0.0043)	11.76% (0.0631)	93.72% (0.0111)	86.76% (0.0609)
	NCHG	2.68% (0.0054)	93.29% (0.0147)	0.00% (0.0000)	82.90% (0.0251)	75.96% (0.0531)
	HGCA	0.76% (0.0016)	93.99% (0.0253)	45.98% (0.1397)	86.04% (0.0340)	82.14% (0.0457)
Stroma	NCLG	0.49% (0.0016)	86.08% (0.0274)	0.48% (0.0034)	24.60% (0.0412)	56.40% (0.0341)
	LGCA	27.73% (0.0581)	76.81% (0.0530)	0.06% (0.0005)	13.97% (0.0133)	74.02% (0.0443)
	NCHG	0.11% (0.0004)	85.05% (0.0265)	0.06% (0.0004)	41.16% (0.0407)	50.06% (0.0630)
	HGCA	33.95% (0.0840)	80.79% (0.0406)	1.29% (0.0077)	49.26% (0.0349)	65.99% (0.0484)
LGCA	Epithelium	p=0.00001 (**)	p=0.1939 (N/S)	p=0.0964 (N/S)	p=0.1635 (N/S)	p=0.8201 (N/S)
	Stroma	p=0.0004 (**)	p=0.1516 (N/S)	p=0.2087 (N/S)	p=0.0199 (*)	p=0.0066 (**)
HGCA	Epithelium	p=0.0045 (**)	p=0.8146 (N/S)	p=0.0063 (**)	p=0.4698 (N/S)	p=0.3931 (N/S)
	Stroma	p=0.0012 (**)	p=0.3942 (N/S)	p=0.1350 (N/S)	p=0.1536 (N/S)	p=0.0975 (N/S)
