# Supplementary material for: Cancer stem cell subpopulations in primary colon adenocarcinoma
Source: PLoS One. 2019 Sep 6;14(9):e0221963. doi: 10.1371/journal.pone.0221963 (PMC6730900; doi:10.1371/journal.pone.0221963)
Supplement: S2 Table — RT-qPCR data showing expression of induced-pluripotent stem cell (iPSC) markers OCT4, SOX2, NANOG, KLF4 and c-MYC. ΔCT values calculated by comparing the gene of interest to housekeeper GAPDH, and ΔΔCT values by comparing high-grade (HG) and low-grade (LG) tumors to their patient-matched normal colon samples. ΔΔCT values used to calculate fold changes using the equation 2^(-ΔΔCT). (PDF) [file pone.0221963.s006.pdf]

S2 Table: RT-qPCR data showing expression of induced-pluripotent stem cell markers

iPSC Markers		ΔCT (tumor)	ΔCT (normal)	ΔΔCT (T-N)	2^(-ΔΔCT)
**OCT4**	LG1	10.75333	10.27334	0.47999	0.717
	LG2	9.1	9.71333	-0.61333	1.53
	LG3	12.5	13.3	-0.8	1.741
	LG4	10.8	9.78334	1.01666	0.494
	LG5	10.88666	9.85666	1.03	0.49
	LG6	10.62333	8.86	1.76333	0.295
	HG1	16.41667	14.39	2.02667	0.245
	HG1	8.92	10.15667	-1.24667	2.373
	HG3	15.19667	13.58667	1.61	0.328
	HG4	11.16334	0	N/A	∞
	HG5	9.15	10.13333	-0.98333	1.977
	HG6	10.01	9.07666	0.93334	0.524
**SOX2**	LG1	5.35333	4.67	0.68333	0.623
	LG2	14.75	14.67	0.08	0.946
	LG3	12.16666	7.69667	4.46999	0.045
	LG4	11.07	4.04	7.03	0.008
	LG5	17.63	15.32	2.31	0.202
	LG6	0	0	N/A	0
	HG1	10.43	9.38334	1.04666	0.484
	HG1	20.17333	16.65667	3.51666	0.087
	HG3	8.3	11.46	-3.16	8.938
	HG4	16.40667	0	N/A	∞
	HG5	0	16.29666	N/A	0
	HG6	16.60333	0	N/A	∞
**NANOG**	LG1	10.93667	13.56	-2.62333	6.162
	LG2	10.75666	11.38666	-0.63	1.548
	LG3	12.82	14.17334	-1.35334	2.555
	LG4	8.97666	10.09667	-1.12001	2.173
	LG5	13.58666	11.09	2.49666	0.177
	LG6	11.52	9.74	1.78	0.291
	HG1	14.67667	11.82667	2.85	0.139
	HG1	8.58	8.68667	-0.10667	1.077
	HG3	14.20667	14.52	-0.31333	1.243
	HG4	10.02	8.09	1.93	0.262
	HG5	8.31667	9.06667	-0.75	1.682
	HG6	8.51	8.58666	-0.07666	1.055
**KLF4**	LG1	5.71333	8.74334	-3.03001	8.168
	LG2	10.70666	8.42333	2.28333	0.205
	LG3	10.81	8.89667	1.91333	0.265
	LG4	11.26666	11.00667	0.25999	0.835
	LG5	8.84	7.05	1.79	0.289
	LG6	8.41666	5.55667	2.85999	0.138
	HG1	12.61333	8.73667	3.87666	0.068
	HG1	11.00667	9.34333	1.66334	0.316
	HG3	12.19	11.77334	0.41666	0.749
	HG4	10.57334	7.64	2.93334	0.131
	HG5	9.26666	11.80333	-2.53667	5.802

	HG6	9.73	8.00333	1.72667	0.302
c-MYC	LG1	6.10333	11.71334	-5.61001	48.841
	LG2	4.4	5.75333	-1.35333	2.555
	LG3	4.09333	3.31	0.78333	0.581
	LG4	7.88333	7.72	0.16	0.895
	LG5	7.76	4.90666	2.85334	0.138
	LG6	6.8	9.25	-2.45	5.464
	HG1	10.26667	8.34667	1.92	0.264
	HG1	2.31667	5.52	-3.20333	9.211
	HG3	8.04333	8.81	-0.76667	1.701
	HG4	4.86	7.08334	-2.22334	4.67
	HG5	4.59	6.22667	-1.63667	3.109
	HG6	4.96667	5.49	-0.52333	1.437
