# Supplementary material for: Cancer stem cell subpopulations in primary colon adenocarcinoma
Source: PLoS One. 2019 Sep 6;14(9):e0221963. doi: 10.1371/journal.pone.0221963 (PMC6730900; doi:10.1371/journal.pone.0221963)
Supplement: S3 Table — Data showing the percentage of cells with mRNA expression of each induced-pluripotent stem cell (iPSC) marker in the epithelium and in the stoma, with the standard error values in brackets. LGCA, low-grade colon adenocarcinoma tissue samples; HGCA, high-grade colon adenocarcinoma tissue samples. NCLG, normal colon tissue from patients with LGCA; NCHG, normal colon tissue from patients with HGCA. Significance values for comparisons between LGCA and HGCA tissue samples and their patient-matched normal colon tissues, for cells in the epithelium and those in the stroma: a p-value between 0.05 and 0.01 is shown by *, and <0.01 represented by **. (PDF) [file pone.0221963.s007.pdf]

S3 Table: *In-situ* Hybridization cell counting data

		iPSC Markers				
		OCT4	SOX2	NANOG	KLF4	c-MYC
Epithelium	NCLG	57.25% (0.0579)	13.47% (0.0200)	57.12% (0.0745)	81.10% (0.0426)	67.12% (0.0490)
	LGCA	76.28% (0.0749)	26.25% (0.0390)	83.18% (0.0318)	64.95% (0.0839)	94.52% (0.0156)
	NCHG	42.73% (0.0263)	12.82% (0.0213)	58.15% (0.0412)	77.47% (0.0541)	70.00% (0.1059)
	HGCA	60.40% (0.1314)	15.58% (0.0303)	65.13% (0.0406)	65.08% (0.0408)	80.60% (0.0807)
Stroma	NCLG	14.66% (0.0371)	7.10% (0.0138)	23.43% (0.0559)	18.50% (0.0316)	14.55% (0.0295
	LGCA	31.52% (0.0467)	9.42% (0.0155)	32.80% (0.0557)	26.47% (0.0328)	35.68% (0.0663)
	NCHG	11.37% (0.0422)	7.05% (0.0130)	26.03% (0.0500)	19.32% (0.0609)	14.35% (0.0230)
	HGCA	29.08% (0.0501)	10.08% (0.0222)	30.73% (0.0633)	21.11% (0.0396)	27.63% (0.0444)
LGCA	Epithelium	p=0.0721 (N/S)	p=0.0154 (*)	p=0.0092 (**)	p=0.1168 (N/S)	p=0.0003 (**)
	Stroma	p=0.0179 (N/S)	p=0.2906 (N/S)	p=0.2627 (N/S)	p=0.1111 (N/S)	p=0.0155 (*)
HGCA	Epithelium	p=0.2530 (N/S)	p=0.4719 (N/S)	p=0.2848 (N/S)	p=0.0975 (N/S)	p=0.2739 (N/S)
	Stroma	p=0.0222 (*)	p=0.2650 (N/S)	p=0.5730 (N/S)	p=0.8093 (N/S)	p=0.0240 (*)
